# Combining Selective Enrichment and a Boosting Approach to Globally and Site-Specifically Characterize Protein Co-translational *O*-GlcNAcylation

**DOI:** 10.1021/acs.analchem.2c04779

**Published:** 2023-02-20

**Authors:** Senhan Xu, Kejun Yin, Ronghu Wu

**Affiliations:** School of Chemistry and Biochemistry and the Petit Institute for Bioengineering and Bioscience, Georgia Institute of Technology, Atlanta, Georgia 30332, United States

## Abstract

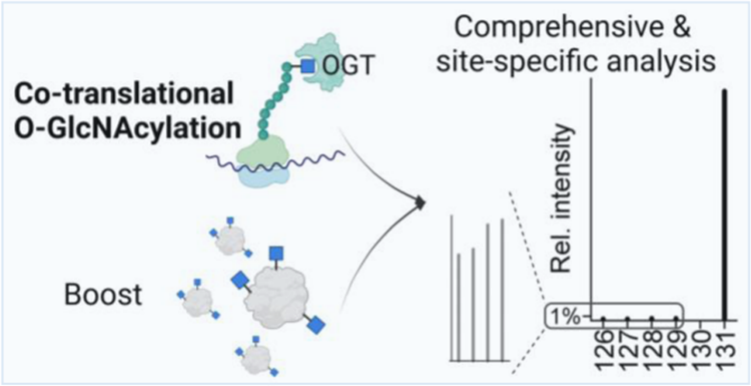

Protein *O*-GlcNAcylation plays extremely important roles in mammalian cells, regulating signal transduction and gene expression. This modification can happen during protein translation, and systematic and site-specific analysis of protein co-translational *O*-GlcNAcylation can advance our understanding of this important modification. However, it is extraordinarily challenging because normally *O*-GlcNAcylated proteins are very low abundant and the abundances of co-translational ones are even much lower. Here, we developed a method integrating selective enrichment, a boosting approach, and multiplexed proteomics to globally and site-specifically characterize protein co-translational *O*-GlcNAcylation. The boosting approach using the TMT labeling dramatically enhances the detection of co-translational glycopeptides with low abundance when enriched *O*-GlcNAcylated peptides from cells with a much longer labeling time was used as a boosting sample. More than 180 co-translational *O*-GlcNAcylated proteins were site-specifically identified. Further analyses revealed that among co-translational glycoproteins, those related to DNA binding and transcription are highly overrepresented using the total identified *O*-GlcNAcylated proteins in the same cells as the background. Compared with the glycosylation sites on all glycoproteins, co-translational sites have different local structures and adjacent amino acid residues. Overall, an integrative method was developed to identify protein co-translational *O*-GlcNAcylation, which is very useful to advance our understanding of this important modification.

## Introduction

*O*-GlcNAcylation occurs on the serine and threonine residues of proteins, and it is the only known type of protein glycosylation primarily occurring in the nucleus and the cytoplasm of mammalian cells.^1,2^ For this dynamic modification, *O*-GlcNAc is added by *O*-GlcNAc transferase (OGT) and is removed by *O*-GlcNAcase (OGA).^3,4^*O*-GlcNAcylation can regulate protein translocation, stability, and interactions with other molecules.^5,6^ To date, thousands of *O*-GlcNAcylation sites in metazoans have been identified, and it was revealed that *O*-GlcNAcylation can play many critical roles in cells, including regulating gene transcription and signal transduction.^7,8^ Recently, *O*-GlcNAcylation was found to occur co-translationally. For example, protein SP1 can be co-translationally *O*-GlcNAcylated, and the stability of the nascent polypeptides was increased by preventing its ubiquitination. Additionally, *O*-GlcNAcylation also stabilized mature SP1.^9^ However, it is still unknown whether *O*-GlcNAcylation stabilizes nascent polypeptides by directly blocking the recognition of ubiquitin ligase or mediating the proper folding of the nascent chains so that they are not degraded through the proteasomal pathway. To further understand this important modification, it is of great interest to investigate protein co-translational *O*-GlcNAcylation at the proteome level.

Previously, co-translationally *O*-GlcNAcylated proteins were labeled with *O*-propargyl-puromycin (OPP) and tetra-*O*-acetyl-2-*N*-azidoacetyl-2-deoxy-d-galactopyranose (Ac_4_GalNAz), followed by sequential enrichments.^10^ Using this method, some co-translationally *O*-GlcNAcylated proteins were identified. Given the importance of this co-translational modification, it is imperative to pinpoint the modification site-specifically for further investigation of its roles in the interactions with other modifications such as phosphorylation and ubiquitination, and the regulation of protein aggregation and degradation. However, the abundances of *O*-GlcNAcylated proteins are very low, and co-translationally *O*-GlcNAcylated proteins have even much lower abundance in cells, making them extraordinarily challenging to be investigated.^11,12^

In this work, we designed an integrative method combining selective enrichment, a boosting approach, and multiplexed proteomics to globally and site-specifically characterize protein co-translational *O*-GlcNAcylation. First, we employed a pulse-chase method to label newly synthesized polypeptides in cells for a short period of time using heavy amino acids. At the same time, puromycin (Puro) and Ac_4_GalNAz were added to the media. Puro was employed to terminate protein synthesis, resulting in the premature release of newly synthesized polypeptides from the ribosome. Ac_4_GalNAz was used to label newly synthesized polypeptides with co-translational *O*-GlcNAcylation. More importantly, due to the low-abundance nature of co-translational glycopeptides compared with many other highly abundant proteins, a boosting approach was applied to enhance the detection of co-translational glycopeptides by liquid chromatography–mass spectrometry (LC–MS). The boosting sample contains *O*-GlcNAcylated peptides enriched from the whole-cell lysate labeled with Ac_4_GalNAz for a much longer time. Furthermore, to prevent the loss of co-translational *O*-GlcNAcylated peptides during sample preparation, we employed the SP3 sample preparation method that retains glycopeptides during sample cleanup.^13,14^ To identify co-translational glycopeptides, the boosting and co-translational samples were labeled with different channels of TMT reagents, respectively, before the samples were mixed. The boosting approach dramatically increased the signal intensities of glycopeptides and facilitated the detection of co-translational *O*-GlcNAcylation. Using this integrative method, we site-specifically identified more than 180 co-translational *O*-GlcNAcylated proteins. Compared with all *O*-GlcNAcylated proteins identified from the same cells, co-translational glycoproteins were found to be more enriched in the nucleus and be involved in transcription. Many co-translational glycoproteins are chromatin regulators and transcription factors. Additionally, the local environments and structures of co-translational *O*-GlcNAcylation sites are significantly different from those of total *O*-GlcNAcylation sites, which is a combination of co-translational and post-translational sites, suggesting the potential preference for the local structures and adjacent amino acid residues of co-translational *O*-GlcNAcylation sites. Overall, we developed an effective method to systematically and site-specifically identify protein co-translational *O*-GlcNAcylation, which provides unprecedented and valuable information for further understanding the functions of protein *O*-GlcNAcylation.

## Experimental Section

### Cell Culture, Metabolic Labeling, and Click Chemistry

MCF7 cells (from the American Type Culture Collection, ATCC) were grown in DMEM medium (Sigma-Aldrich) containing 10% fetal bovine serum (FBS, Thermo) at 37 °C with 5.0% CO_2_ in a humidified incubator. When the confluency reached 80%, the medium was replaced using a heavy lysine (K8) and arginine (R6) containing medium, and 250 μM *N*-azidoacetylgalactosamine-tetraacetylated (Ac_4_GalNAz, Click Chemistry Tools) and 50 μM puromycin (Puro, Santa Cruz Biotechnology) were added to the medium. For the control samples, Puro was not added. Then, cells were treated for 1 h. For the boosting sample, cells were cultured in the medium containing heavy lysine and arginine for 2 weeks for complete labeling of proteins with heavy K and R in the cells. Then the cells were treated with 250 μM Ac_4_GalNAz for 48 h to label *O*-GlcNAcylated proteins.

Cells from different samples were harvested and washed with ice-cold PBS twice. They were lysed with a buffer containing 50 mM HEPES, pH = 7.4, 150 mM NaCl, 0.5% SDC, 0.1% SDS, 1% NP-40, 50 μM Thiamet G, 50 units/mL Benzonase nuclease (Millipore), and 1 tablet/10 mL EDTA-free protease inhibitor for 2 h at 4 °C. Then the lysates were centrifuged for 10 min at 4696*g*, and the debris was discarded. The labeled glycopeptides or glycoproteins were reacted with a biotin probe through a click chemistry reaction. In the cell lysate, 250 μM photocleavable (PC) biotin-alkyne (Click Chemistry Tools), 1 mM CuSO_4_, 5 mM Tris(3-hydroxypropyltriazolylmethyl) amine (THPTA, Click Chemistry Tools), 5% dimethyl sulfoxide (DMSO), 15 mM sodium l-ascorbate (Sigma), and 15 mM aminoguanidine hydrochloride (Sigma) were added, and the reaction lasted for 2 h at room temperature.

### SP3 Sample Cleanup and Glycoprotein Enrichment

The paramagnetic beads, i.e., Sera-Mag SpeedBead Carboxylate-Modified [E3] Magnetic Particles (Cytiva) and Sera-Mag SpeedBead Carboxylate-Modified [E7] Magnetic Particles (Cytiva), were used for sample cleanup. The beads were washed with water twice before use. The beads were added to the lysate in a ratio of 10:1 (wt/wt, beads to proteins) based on the well-established protocol.^15^ In the bead–lysate mixture, acetonitrile (ACN) was slowly added with constant swirling to ensure that the beads did not stick to the conical tube wall until ACN reached a final concentration of 95%. The mixture was incubated at 37 °C for 20 min to maximize the binding of peptides and proteins to the beads. Then the tubes were placed in magnetic racks, and the unbound supernatant was removed after the beads migrated to the tube wall. The beads were washed with 95% ACN three times. A digestion buffer containing 50 mM HEPES, pH = 8.6, 1.6 M urea, and sequence grade trypsin (Promega) was added, followed by incubation for 16 h at 37 °C to digest proteins. The peptide solutions were acidified and desalted using the tC18 Sep-Pak cartridges (Waters). Glycopeptides were enriched using NeutrAvidin resins in PBS at room temperature for 1 h. The resins were washed with PBS for eight times and water for two times to remove nonspecific binding peptides. The enriched glycopeptides were eluted twice under UV radiation, each for 1 h at room temperature.

### TMT Labeling and Peptide Fractionation

The enriched glycopeptides from different samples were labeled with the TMT sixplex (Thermo) reagents in 200 mM HEPES, pH = 8.6. The duplicate samples with co-translational *O*-GlcNAcylated peptides were labeled with the first two channels (126 and 127), and the duplicate control experiments were labeled with the channels of 128 and 129. The boosting sample was labeled with the channel of 131. The channel of 130 was not used to avoid potential isotopic contamination from the boosting sample. For the control experiment without the boosting approach, no boosting sample was used, and all of the other experimental setups were the same. The reaction lasted for 1 h at room temperature, and then it was quenched by adding hydroxylamine hydrochloride (Sigma). The labeled glycopeptides were purified using a stage-tip. Then, the glycopeptides were fractionated through being sequentially eluted with different concentrations of ACN with 1% of acetic acid before being analyzed by liquid chromatography coupled to tandem mass spectrometry (LC–MS/MS).

### LC–MS/MS Analysis

The peptides were resuspended in a solution containing 5% ACN and 4% FA, and 4 μL was loaded to a Dionex WPS-3000TPLRS autosampler (UltiMate 3000 thermostatted Rapid Separation Pulled Loop Wellplate Sampler) equipped with a microcapillary column packed with C18 beads (Magic C18AQ, 3 μm, 200 Å, 75 μm × 16 cm, Michrom Bioresources). Peptides were separated by reversed-phase high-performance liquid chromatography (HPLC) using an UltiMate 3000 binary pump with 120 min gradients of 1–20% or 2–32% ACN (with 0.125% FA) and were analyzed by a hybrid dual-cell quadrupole linear ion trap–Orbitrap mass spectrometer (LTQ Orbitrap Elite, Thermo Fisher). The data-dependent acquisition mode with the Top15 method was used for precursor selection with a resolution of 60,000, and the resolution for tandem MS was 15,000. The selected ions were excluded from further sequencing in 90 seconds. The normalized collision energy (NCE) was set at 34% to fragment precursor ions.

### Database Search and Data Filtering

The raw files were converted into mzXML files and searched against the human (*Homo sapiens*) proteome database from UniProt using the SEQUEST algorithm (version 28).^16^ The following parameters were used for the search of glycopeptides: 20 ppm precursor mass tolerance; 0.025 Da fragment ion mass tolerance; up to two missed cleavages; up to three modifications on each peptide; variable modification on serine, threonine, and cysteine (+528.2859 Da) for TMT-labeled *O*-GlcNAc; and fixed modifications including oxidation of methionine (+15.9949 Da) and TMT modification on lysine and the peptide N-terminus (+229.1629 Da). For glycopeptide identifications, the data quality was evaluated and well-controlled using linear discriminant analysis (LDA).^17^ The parameters used include XCorr, ΔCorr, missed cleavages, adjusted ppm, peptide length, and charge state. Peptides with fewer than seven amino acid residues were discarded. Finally, the false discovery rates (FDRs) of glycopeptides were controlled to <1%. ModScore was used to determine the confidence of the glycosylation site localization.^18^ Sites with ModScore >13 (*P* < 0.05) were considered as confidently localized, and only the confidently localized sites were used for site-specific analysis.^19,20^ To remove *S*-glycosylation sites, we have applied very stringent criteria to make sure that only confidently identified *O*-glycosylation sites were included in the data set. The detailed filtering steps are the same with our previous publications.^6,20^ The isotopic correction parameters provided by Thermo were used to calibrate the TMT reporter ion intensities before data analysis. For the two TMT channels for the samples of co-translational *O*-GlcNAcylation, the *S*/*N* ratio in at least one sample has to be >5 for confident annotation of co-translational *O*-GlcNAcylated peptides.

### Bioinformatic Analysis

Protein annotation information was analyzed using the Database for Annotation, Visualization, and Integrated Discovery (DAVID, https://david.ncifcrf.gov/).^21^ Only well-localized sites with the ModScore value of >13 were selected to perform the motif analysis using the online software pLogo (https://plogo.uconn.edu/).^22^ The protein interaction information was extracted from the String database and visualized by Cytoscape. Proteins were further grouped based on their GO annotations.^23,24^ Protein abundance information was obtained from the PAXdb database.^25^ The information of protein domains was extracted from the InterPro database,^26^ UniProt database, and the online prediction software SUPERFAMILY.^27^ The information for protein physiochemical properties was extracted from the R package “Peptides” (https://github.com/dosorio/Peptides/). Ubiquitination and phosphorylation sites were found from the online web site of PhosphoSitePlus (https://www.phosphosite.org).^28^ Protein secondary structures, solvent accessibility, and structural disorder were predicted by RaptorX.^29^

## Results

### Development of a Method to Identify Protein Co-translational *O*-GlcNAcylation Sites in Human Cells

As *O*-GlcNAcylated proteins are normally of low abundance in cells, it is very challenging to detect *O*-GlcNAcylation sites without enrichment. To enrich *O*-GlcNAcylated proteins, a sugar analogue containing an azide group was used to label glycoproteins. The sugar analogue is structurally similar to its natural counterpart and can be incorporated into glycoproteins.^30−33^ In this work, Ac_4_GalNAz was used as it has been widely applied to label *O*-GlcNAcylated proteins.^34−36^ The azide group enables further enrichment of labeled glycoproteins using click chemistry. Moreover, newly synthesized glycoproteins labeled with Ac_4_GalNAz carrying an azide group are different from existing glycoproteins, ensuring only newly synthesized *O*-GlcNAcylated proteins/peptides being enriched and analyzed. To analyze protein co-translational *O*-GlcNAcylation, we adopted a pulse-chase method, i.e., the cell culture medium with light amino acids being switched to the medium containing isotopically labeled heavy lysine and arginine (K8 and R6). Due to the mass difference, MS can easily distinguish newly synthesized proteins from existing ones.

**Figure 1:**
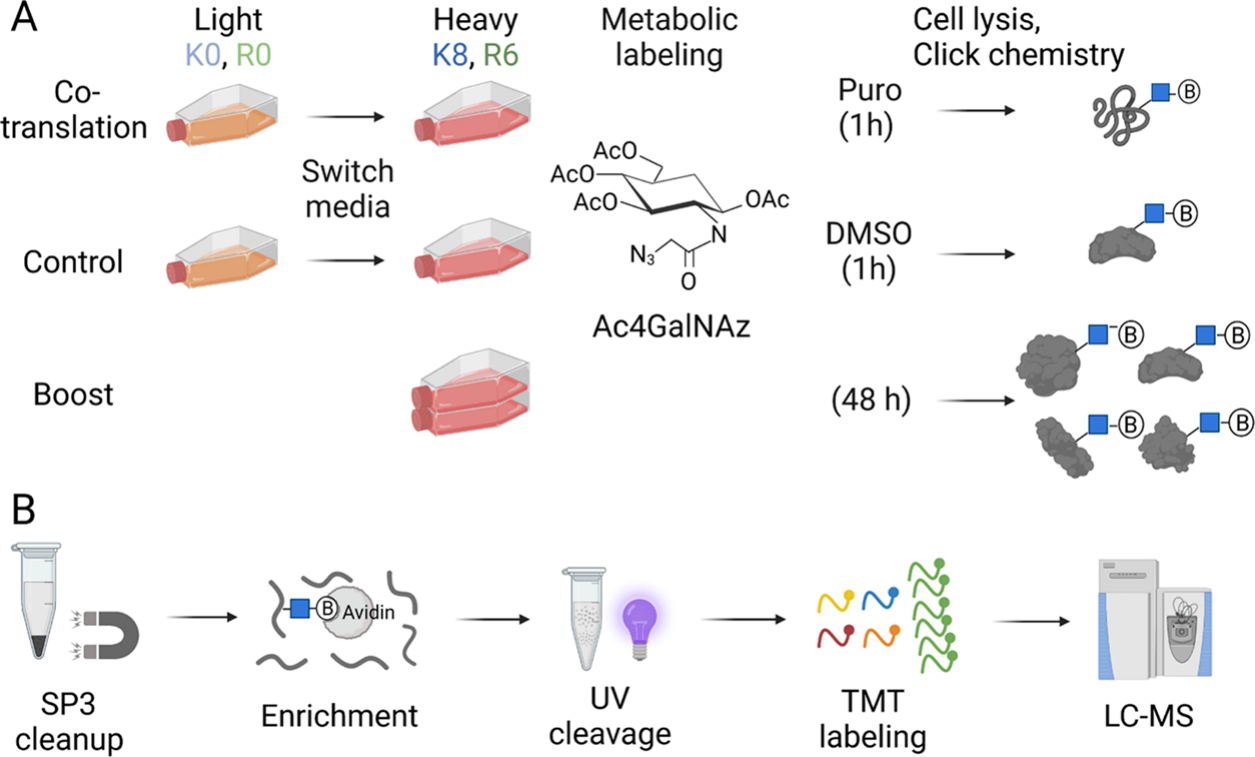
(A) Experimental workflow for the labeling of co-translational *O*-GlcNAcylation and boosting samples. In the boosting sample, *O*-GlcNAcylated proteins were labeled with Ac_4_GalNAz for 48 h in heavy media. For the co-translational and control samples, the culture media were switched from light to heavy, and cells were further cultured for 1 h with Ac_4_GalNAz. At the same time, Puro was added to the co-translational samples while DMSO was added to the control samples. Then the cells were lysed, and click chemistry was performed in the cell lysate to add a photocleavable biotin tag to labeled glycoproteins. (B) Experimental workflow for the enrichment of glycopeptides. The biotin-labeled glycopeptides were enriched using NeutrAvidin resin and eluted under UV radiation. The boosting, co-translational, and control samples were labeled with the TMT reagents, respectively, before being mixed. The samples were fractionated and analyzed by LC–MS/MS.

Puro is an aminonucleoside antibiotic that resembles the 3′ end of the aminoacylated tRNA. During protein synthesis, it enters the ribosomal A site and is added to the polypeptide chain. Once modified by Puro, the synthesis is terminated and the polypeptide is released from the ribosome possibly because the amide bond at the 3′ position is resistant to hydrolysis.^37^ Therefore, Puro was used to prevent the generation of mature proteins.^38^ It was added simultaneously with Ac_4_GalNAz and heavy amino acids to the cell culture medium for labeling co-translationally *O*-GlcNAcylated polypeptides (Figure 1A). Additionally, the sample without the Puro treatment was used as a control. Because a longer treatment time can result in the maturation of some proteins and different protein expressions due to cell response to the Puro treatment, the cells were only treated for a short period of time (1 h) with Puro and the sugar analogue in the heavy culture medium. However, due to the short labeling time and the protein synthesis inhibition by Puro, very limited amount of co-translational *O*-GlcNAcylated polypeptides are produced, and thus, many of them are below the detection limit of MS, which prevents global analysis of protein co-translational *O*-GlcNAcylation. To overcome this issue, a boosting approach was employed to enhance the detection of co-translational glycopeptides. A boosting sample labeled with Ac_4_GalNAz in the heavy culture medium for 48 h contained many more copies of *O*-GlcNAcylated proteins. The incorporation of the boosting channel can significantly enhance the signals of glycopeptides at both MS1 and MS2 levels, resulting in massive improvement in the identification of co-translational glycopeptides.^39,40^

Cells from different samples were lysed, and then photocleavable (PC) biotin-alkyne was added to react with labeled glycopeptides through click chemistry. As the Puro treatment caused incomplete synthesis of nascent glycoproteins, generally co-translational *O*-GlcNAcylated proteins may be truncated and have relatively smaller sizes. To avoid the potential sample loss during protein precipitation, which is ineffective in precipitating small proteins, the SP3 cleanup method was employed to purify proteins in the cell lysate.^41,42^ SP3 is a solid-phase enhanced sample preparation method relying on hydrophilic interactions between proteins and the coated paramagnetic beads, while detergents, salts, and solvents are removed through washing. Peptides can effectively bind to the beads when the concentration of the organic solvent reaches 95%, which enables the purification of smaller newly synthesized proteins due to the Puro treatment. Then glycopeptides were enriched using the NeutrAvidin resins and specifically released under UV radiation. Next, the samples were labeled with the TMT reagents before being mixed. TMT is a set of chemical labeling reagents that react with the amine group on peptides. The reagents are isotopically coded and have the same molecular weight. In MS1, the same glycopeptides from different samples will be picked simultaneously for their sequencing. In MS2, the resulted reporter ions with different masses from the peptides in different samples allow us to accurately quantify them. When the boosting approach was used, the boosting sample with Ac_4_GalNAz labeling for a much longer time (48 h) and the co-translational *O*-GlcNAcylation samples were labeled with the TMT reagents, respectively, and mixed together. The boosting sample dramatically enhanced the detection of co-translational glycopeptides at both the MS1 and MS2 levels.

### Identification of Co-translational *O*-GlcNAcylation Sites in Human Cells

**Figure 2:**
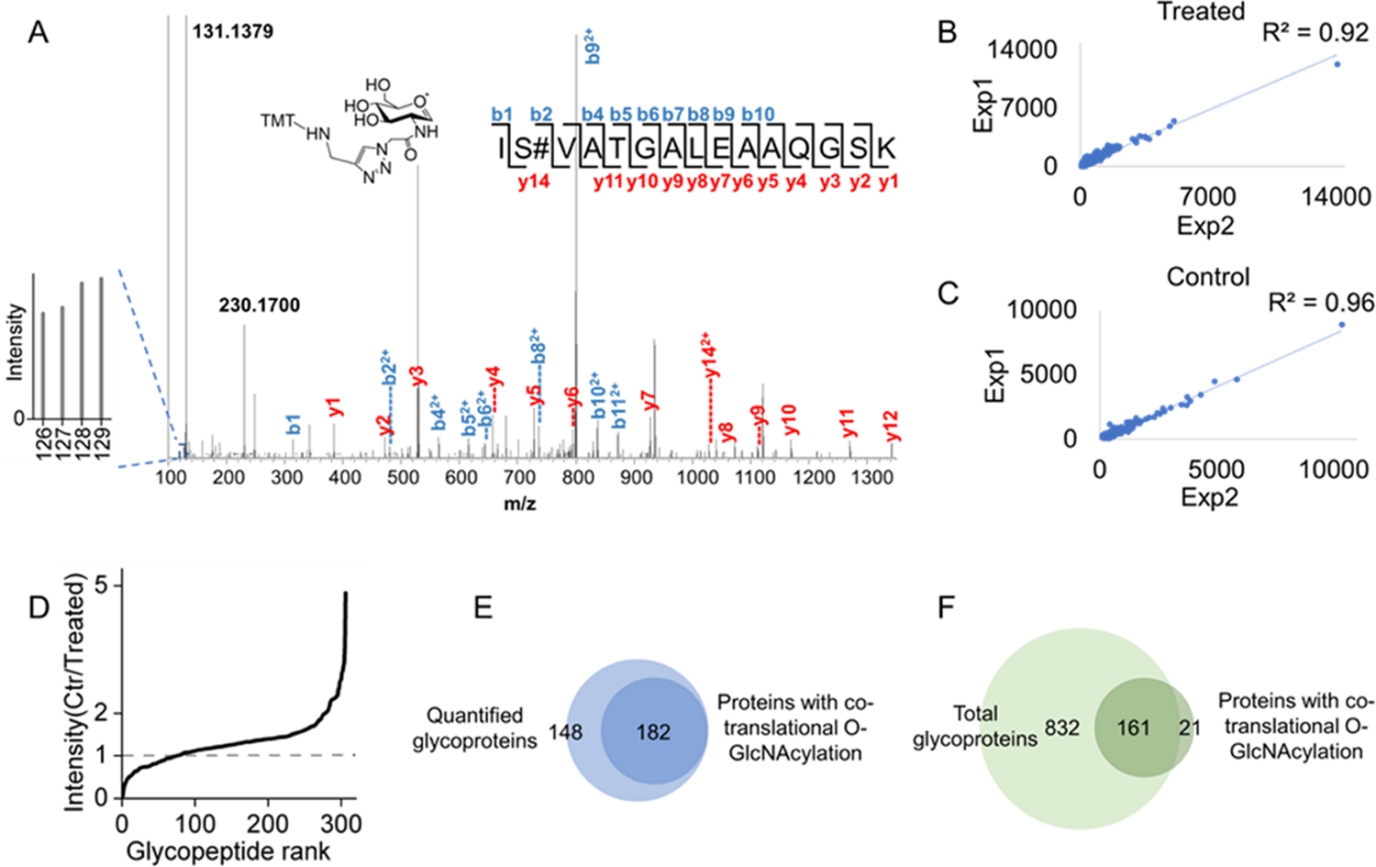
(A) Example tandem mass spectrum of an identified co-translational *O*-GlcNAcylated peptide. (B) Correlations of the quantified *O*-GlcNAcylated peptides with the Puro treatment in the duplicate experiments. (C) Correlations of the quantified *O*-GlcNAcylated peptides in the duplicate control experiments. (D) Ratios of the intensities of the unique co-translational *O*-GlcNAcylated peptides in the treated samples vs the control ones. (E) Venn diagram of co-translational glycoproteins among all quantified *O*-GlcNAcylated proteins. (F) Comparison of co-translational glycoproteins with all *O*-GlcNAcylated proteins in MCF7 cells identified in another experiment.

An example spectrum for a TMT-labeled glycopeptide is shown in Figure 2A. The glycopeptide was confidently identified with XCorr = 3.23 and the glycosylation site was well-localized with ModScore = 65.08. The presence of the glycan on the peptide was further proven by the fragment ion at 529.2930 (*m*/*z*), which agrees very well with the mass of the TMT-tagged *O*-GlcNAc (protonated, 529.2937 Da). The peptide is from the protein host cell factor 1 (HCFC1), which has a variety of functions in transcription, and is a known *O*-GlcNAcylated protein.^43^ As expected, the boosting channel has much higher intensity than those from the co-translational *O*-GlcNAc samples, which significantly increases the identification of glycopeptides with co-translational *O*-GlcNAcylation. Among quantified glycopeptides, both the treated and control samples show excellent correlations between the duplicate experiments (*R*^2^ = 0.92 for the treated samples and *R*^2^ = 0.96 for the control ones), indicating that the experiments have high reproducibility (Figure 2B,C).

To demonstrate that the integration of a boosting approach is critical for the identification of co-translational *O*-GlcNAcylation, we performed an experiment without the boosting sample, i.e., all of the experimental conditions are exactly the same except without the sample from the heavy-labeled cells treated with Ac_4_GalNAz for 48 h (Figure S1). From this experiment, we were not able to identify any co-translational glycopeptide. The abundance of co-translational glycopeptides from only 1-hour labeling is extremely low, and thus even with the enrichment, they still cannot be detected. The average intensities of the co-translational samples are less than 1% of that of the boost one (Figure S2A). As co-translational glycopeptides have extremely low abundance, the integration of the boosting approach is imperative for the identification of co-translational glycopeptides by MS.

An extended view of the average intensities from the first four channels is shown in Figure S2B. With the Puro treatment that prematurely terminates the protein synthesis, co-translational *O*-GlcNAcylated peptides have overall lower intensity compared with the control samples. The ratios of glycopeptides in the control samples versus the treated ones indicate that the majority of glycopeptides have higher intensities in the control samples than the corresponding co-translational ones in the Puro-treated samples (Figure 2D). This is because Puro terminated the protein synthesis, resulting in fewer newly synthesized proteins, especially co-translational glycopeptides during the 1-hour labeling.^44^ A small fraction of glycopeptides have higher intensities in the Puro-treated sample, which could be due to the cell response to the Puro treatment.^45^

In total, 330 *O*-GlcNAcylated proteins were quantified. To confidently identify co-translational *O*-GlcNAcylated proteins, it was required that the signal to noise (*S*/*N*) ratio is larger than 5 in at least one sample with the Puro treatment to be considered as a co-translational glycopeptide. Among quantified glycoproteins, 182 proteins are confidently identified co-translational glycoproteins (Figure 2E, Table S1), and 50 co-translational *O*-GlcNAcylated proteins have more than one co-translational glycosylation site identified. To compare the properties of co-translational *O*-GlcNAcylated proteins and sites with all identified *O*-GlcNAcylated proteins, we performed another proteomics experiment to site-specifically identify all *O*-GlcNAcylated proteins in the same type of cells (MCF7 cells). Around 990 *O*-GlcNAcylated proteins were identified (Table S2), and it shows a great overlap with 161 out of 182 co-translational *O*-GlcNAcylated proteins also found among total *O*-GlcNAcylated proteins (Figure 2F).

### Comparison of Co-translational and Total *O*-GlcNAcylated Proteins

**Figure 3:**
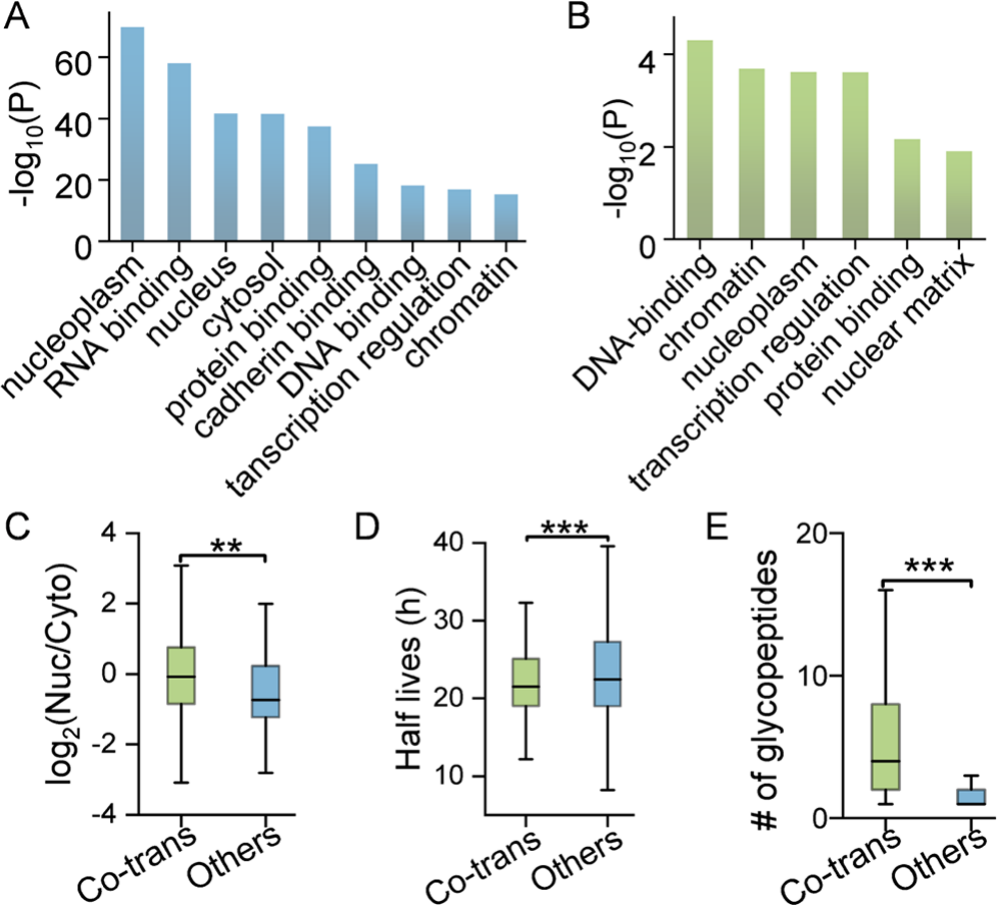
(A) Clustering results for all *O*-GlcNAcylated proteins against the human proteome. (B) Clustering results for co-translational *O*-GlcNAcylated proteins against total *O*-GlcNAcylated proteins identified in MCF7 cells. (C) Comparison of the distribution of the proteins with co-translational glycosylation sites and others in the nucleus (Nuc) and in the cytoplasm (Cyto). (D) Comparison of the dynamics of the proteins identified with co-translational glycosylation sites and others in the nucleus. (E) Number of the glycopeptides identified from each protein between those with co-translational glycosylation sites and others. In the boxplots, the center line indicates the median value, box limits indicate the first and third quartiles, and whiskers indicate 1.5 interquartile range. Statistical significance was determined by the Student’s *t*-test, two tailed. The significance levels are labeled as ** (*P* < 0.01), *** (*P* < 0.001), and **** (*P* < 0.0001).

We compared the functions and distributions between co-translational and total *O*-GlcNAcylated proteins. The total glycoproteins identified in MCF7 cells were clustered based on cellular compartment, molecular function, and biological process (Figure 3A) using the *H. sapiens* proteome as the background. The results are consistent with our previous knowledge about protein *O*-GlcNAcylation. They mostly reside in the nucleus and the cytosol; are involved in DNA, RNA, and protein binding; and regulate protein transcription. Furthermore, using total *O*-GlcNAcylated proteins identified here as the background, co-translational glycoproteins were clustered (Figure 3B). Interestingly, co-translational glycoproteins are highly enriched in the nucleus, particularly in the chromatin, the nucleoplasm, and the nuclear matrix. They are involved in DNA and protein binding and associated with transcription. The results demonstrate that co-translational *O*-GlcNAcylation occurs preferentially on proteins in the nucleus and is more related to transcription.

To further validate this observation, we have extracted the information about the nuclear–cytoplasmic distribution of *O*-GlcNAcylated proteins from our previous work, and compared the distribution of the co-translational *O*-GlcNAcylated proteins with others without co-translational *O*-GlcNAcylation.^6^ The results further verify that co-translational *O*-GlcNAcylated proteins have higher nuclear distribution (Figure 3C). Co-translationally *O*-GlcNAcylated proteins are more dynamic compared with others in the nucleus (Figure 3D). The same trend was also observed in the cytoplasm (Figure S3). Next, we compared the number of glycopeptides identified from glycoproteins with or without co-translational *O*-GlcNAcylation. It was found that glycoproteins with co-translational sites have significantly more glycopeptides identified (Figure 3E), suggesting that co-translational *O*-GlcNAcylation on proteins with higher abundance or higher stoichiometry has a better chance to be detected. Finally, the protein properties, including size, hydrophobicity, and charge, were compared between co-translational and total *O*-GlcNAcylated proteins. Co-translational glycoproteins were found to have significantly higher molecular weight than total *O*-GlcNAcylated ones (Figure S4), which is also consistent with the result above.

### Proteins with Co-translational *O*-GlcNAcylation Associated with Different Localizations and Functions

**Figure 4:**
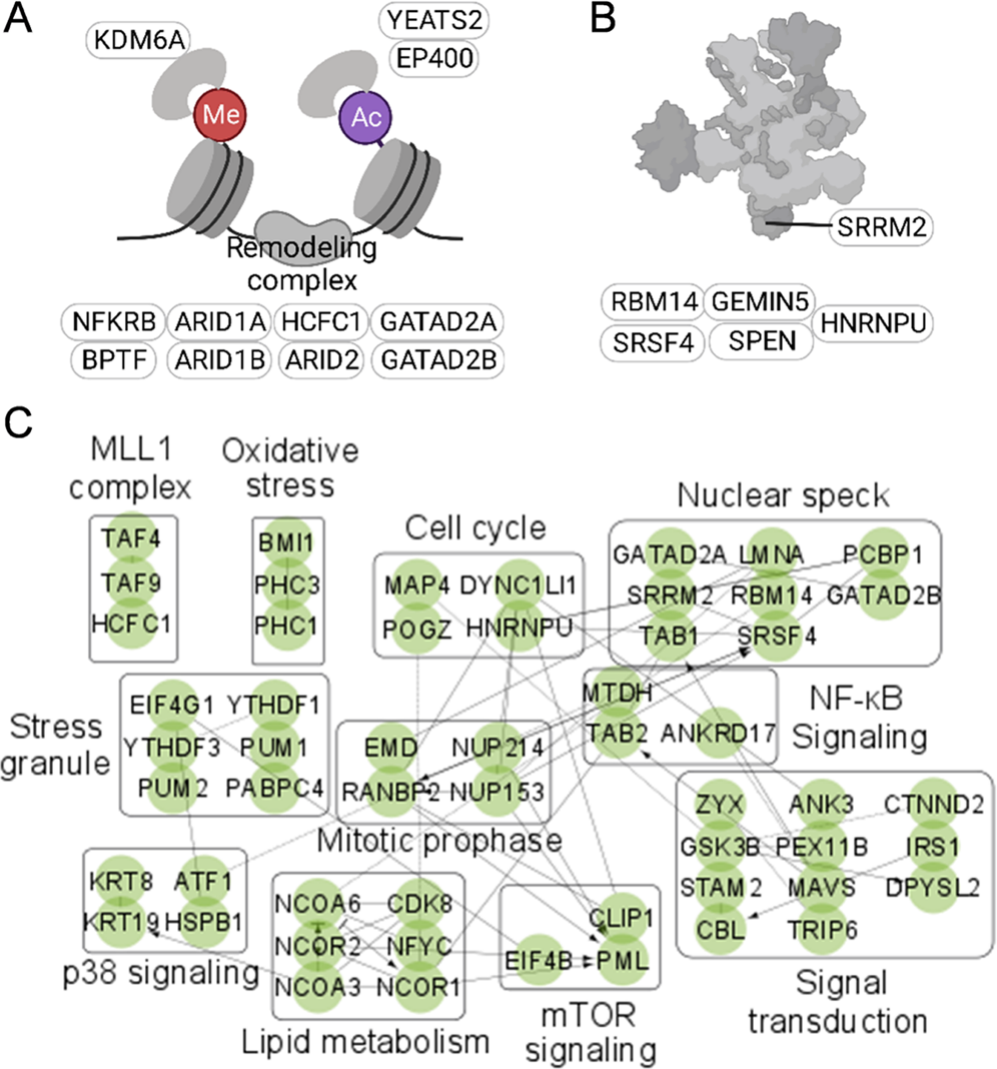
(A and B) Illustrations of the chromatin remodeling complex (A) and the spliceosome (B). (C) Identification of co-translational *O*-GlcNAcylated proteins associated with different protein complexes and biological processes. Each node stands for a protein, and each edge represents a known protein–protein interaction. The arrowheads indicate the direction of interaction from a source node to a target node.

Some examples of co-translational *O*-GlcNAcylated proteins as chromatin regulators are shown in Figure 4A. These glycoproteins are in the histone remodeling complex and may be involved in modulating histone modifications, including acetylation and methylation. Previously, it was reported that *O*-GlcNAcylation could modify many chromatin regulators to affect their stability and functions in transcription.^46,47^ Besides chromatin regulators, six co-translational *O*-GlcNAcylated proteins associated with the spliceosome were also identified (Figure 4B). It was reported that *O*-GlcNAcylation is involved in mRNA splicing. First, *O*-GlcNAcylation controls gene expression through regulating the splicing of the detained introns.^48^ Additionally, *O*-GlcNAcylation of TDP-43 can promote its RNA splicing function.^49^ Conversely, the OGT expression is regulated by intron retention, which dynamically responds to the overall *O*-GlcNAcylation level in cells.^50^ Three proteins in the nuclear pore complex (NPC) were modified with co-translational *O*-GlcNAcylation (NUP214, NUP153, and RANBP2). Among them, NUP214 is a component of the cytoplasmic filament of the NPC and plays an important role in mediating nuclear–cytoplasmic transport.^51^ NUP153 is a part of the nuclear basket required for the anchoring of the NPC and the docking of importing karyopherins.^52^*O*-GlcNAcylation is very important for proteins in the NPC, as it regulates the protein bidirectional transport and the stabilities of proteins in the NPC.^53,54^

Many transcription factors are also *O*-GlcNAcylated, which is important for the regulation of gene expression.^55,56^ In our data set, about 20 transcription factors were found to be co-translationally glycosylated (Table S3), and some examples are listed in Table 1. For example, STAT1 plays a vital role in cellular response to infection and the activation of inflammation.^57^ It was reported that *O*-GlcNAcylation could protect STAT1 from degradation and regulate the protein in response to virus.^58,59^*O*-GlcNAcylation of ELF1 results in its nuclear translocation and binding to the promoter of the TCR ζ-chain.^60^ Additionally, ELF1 interaction with SP1 is inhibited by *O*-GlcNAcylation, which negatively affects the expression of the oncofetal protein gene (Pem).^61^ As *O*-GlcNAcylation is associated with gene expression, it may be very interesting to further study the regulation of gene expression by the co-translational modification.

**Table 1:** Some Example Transcription Factors with Co-translational *O*-GlcNAcylation Identified in This Work^a^

gene name	glycopeptides	XCorr	PPM	site	ModScore	annotation
**TOX4**	GLQLGQTST#ATIQPSQQAQIVTR	6.55	0.34	407	17.4	TOX high mobility group box family member 4
**HCFC1**	T#MAVTPGTTTLPATVK	3.31	0.15	579	31.4	host cell factor C1
**BPTF**	TVIT#EVTTMTSTVATESK	3.69	2.59	1712	15.3	nucleosome remodeling factor subunit
**TAF9**	LSVGSVTSRPSTPTLGT#PTPQTMSVSTK	3.22	–1.98	164	18.3	transcription initiation factor TFIID subunit 9
**ELMSAN1**	AREDSGMVPLIIPVS#VPVR	3.36	–0.20	530	109.0	mitotic deacetylase-associated SANT protein
**ATF7IP**	T#SLPTVGPSGLYSPSTNR	3.09	1.36	887	20.0	activating transcription factor 7-interacting protein 1
**LPP**	S#TGEPLGHVPAR	3.27	2.02	11	20.0	lipoma-preferred partner
**EP400**	AQPAIT#TGGSAAVLAGTIK	3.12	1.62	2604	20.0	E1A-binding protein p400
**ZHX3**	EGDHS#FINGAVPVSQASASSAK	4.25	–0.59	231	138.2	zinc fingers and homeoboxes protein 3

a #denotes the glycosylation site.

We also investigated co-translational *O*-GlcNAcylated proteins in different protein complexes and biological processes (Figure 4C). Several proteins in the stress granules are co-translationally *O*-GlcNAcylated. It is well-known that *O*-GlcNAc is a prominent component of the stress granule, and during heat stress, *O*-GlcNAcylation of eIF4G is critical for the translation of mRNAs for proteins responding to the stress.^62,63^*O*-GlcNAcylation also plays a multifaceted role in the cell cycle, and it regulates gene expression by modifying a large subset of transcription-related proteins and modulates the functions of proteins involved in the spindle apparatus and cytokinesis.^64^ Furthermore, *O*-GlcNAcylation serves as a nutrient sensor that is not only related to glucose metabolism but also involved in the metabolism of other nutrients, including lipids.^65^ Six proteins identified with co-translational *O*-GlcNAcylation are associated with lipid metabolism. Additionally, *O*-GlcNAcylation regulates multiple signaling pathways, making it an excellent signal integrator for a wide range of cellular events.^66^ We have identified co-translational *O*-GlcNAcylation in p38, NF-κB, and mTOR signaling pathways, and it is already known that they are regulated by *O*-GlcNAcylation.^67−69^ The current results demonstrate that a wide array of proteins were co-translationally *O*-GlcNAcylated.

### Local Structures and Adjacent Amino Acid Residues of Co-translational *O*-GlcNAcylation Sites

**Figure 5:**
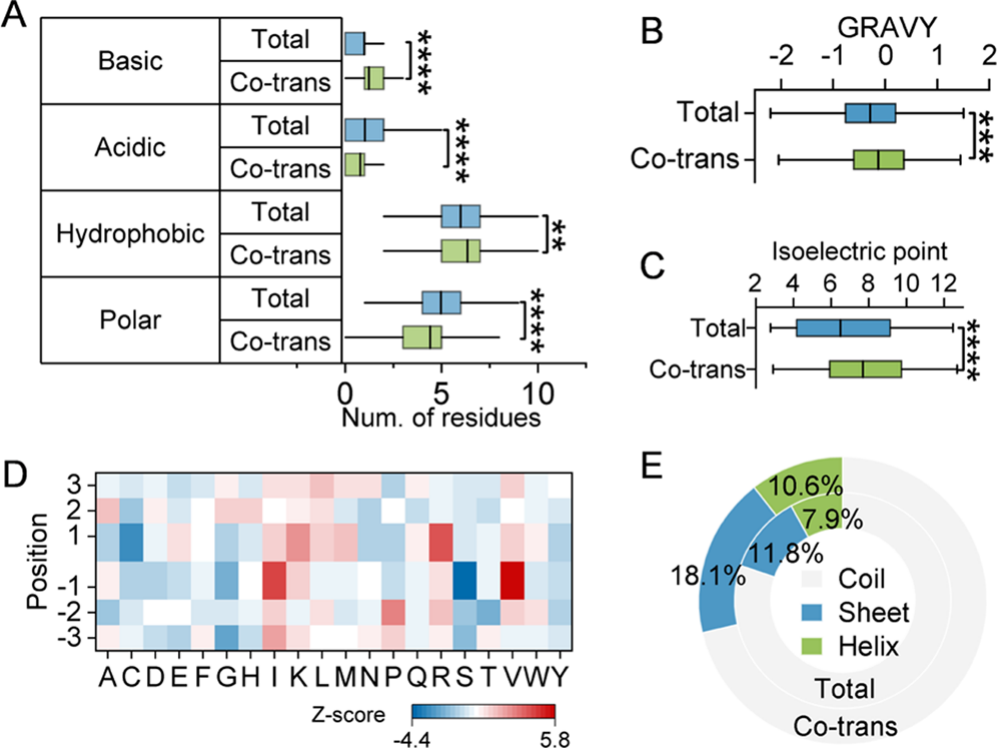
(A–C) Comparison of the amino acid composition (A), the GRAVY score (B), and isoelectric point (C) of the adjacent residues between the co-translational and total *O*-GlcNAcylation sites. (D) Occurrence of different amino acids next to the co-translational *O*-GlcNAcylation sites, using all *O*-GlcNAcylation sites identified in MCF7 cells as the background. The amino acids overrepresented in the co-translational glycosylation sites have positive *Z*-scores, while those reduced have negative ones. (E) Comparison of the secondary structures of the co-translational and total glycosylation sites. In the boxplots, the center line indicates the mean value, box limits indicate the first and third quartiles, and whiskers indicate 1.5 interquartile range. Statistical significance was determined by the Student’s *t*-test, two tailed. The significance levels are labeled as ** (*P* < 0.01), *** (*P* < 0.001), and **** (*P* < 0.0001).

To further understand co-translational *O*-GlcNAcylation, the well-localized co-translational *O*-GlcNAcylation sites (Table S4) were compared with total glycosylation sites (Table S5) in MCF7 cells to further assess their differences. The adjacent (±6) amino acid residues of the glycosylation sites were extracted, and the 13-mer for each glycosylation site was constructed. It was found that the compositions of the nearby residues are dramatically different. The co-translational glycosylation sites have significantly more basic and hydrophobic amino acid residues, but fewer acidic and polar residues nearby compared with total glycosylation sites (Figure 5A).

The GRAVY (average hydrophobicity) score for the 13-mers of the co-translational and total glycosylation sites was calculated and compared (Figure 5B), and those co-translationally modified ones are on average more hydrophobic. The average isoelectric point for co-translational ones is much higher than that of the total ones: close to 8 vs slightly above 6 (Figure 5C). The occurrence of different amino acids next to the co-translational glycosylation sites was generated against the background of all *O*-GlcNAcylation sites identified in MCF7 cells (Figure 5D). Arginine is dramatically overrepresented at +1 position, while valine and isoleucine are enriched at −1 position. These results demonstrate that some *O*-GlcNAcylation sites are preferentially modified during the translation due to the adjacent sequences likely favored by OGT. OGT was reported to be preferentially bound to β-branched residues (I, V, and T) in positions flanking the glycosylation sites.^70^ Additionally, the α-phosphate of UDP-GlcNAc may interact with basic residues to enhance the binding of the peptide backbone when glycosylation happens.^71^ These results indicate that the local environments of co-translational *O*-GlcNAcylation sites have some difference from the other ones. This may be because OGT has the preference for binding with some amino acid residues nearby glycosylation sites on nascent polypeptides that facilitates the co-translational glycosylation process.

It was reported that protein co-translational *O*-GlcNAcylation can prevent nascent polypeptides from degradation through ubiquitination.^9^ Therefore, co-translational *O*-GlcNAcylation may play an important role in regulating the stability of newly synthesized polypeptides. Phosphorylation and ubiquitination sites next to the glycosylation sites (±10 amino acid residues) were extracted from PhosphositePlus.^28^ The co-translational sites were found to have a slightly higher chance of being in proximity of phosphorylation or ubiquitination sites, which is in accordance with the previous observation that co-translational *O*-GlcNAcylation can prevent ubiquitination to regulate polypeptide degradation (Figure S5). Finally, we investigated the secondary structures near the glycosylation sites, and the co-translational glycosylation sites were highly likely to reside in sheet and helix, but less frequently in coil (Figure 5E). Similarly, the co-translational sites are more probably found in ordered regions and are less likely exposed on the protein surface (Figure S6). Co-translational *O*-GlcNAcylation occurs when nascent peptides are not well-folded, resulting in a higher possibility of modifying the buried and ordered regions, which are less accessible after proteins are properly folded.

## Discussion

During protein translation, different processing and modifications are involved to control the protein quality and regulate the stability of newly synthesized polypeptides. For example, methionine aminopeptidase (MAP) can cleave the N-terminal methionine, resulting in the exposure of a variety of amino acids at the N-termini.^72^ Furthermore, the N-termini of nascent polypeptides can be modified with different types of acylation such as propionylation and myristoylation, and the most prominent one is acetylation, which is widely regarded as the stabilizing signal to prevent proteins from degradation.^73,74^ Co-translational ubiquitination was reported to be responsible for the swift degradation of ∼15% newly synthesized proteins in cells to remove misfolded proteins. In the ER lumen, nascent proteins are *N*-glycosylated at the asparagine residues by the STT3A complex, and *N*-glycosylation is critical for the proper folding and transport of proteins.^75,76^ Recently, it was found that *O*-GlcNAcylation can also happen co-translationally, which may prevent the degradation of nascent polypeptides. However, many questions are yet to be answered: How often does co-translational *O*-GlcNAcylation happen? On which proteins and sites does co-translational *O*-GlcNAcylation occur? What are the functions of this co-translational modification?

To further study this co-translational modification, there is an urgent need to identify co-translationally *O*-GlcNAcylated proteins and to pinpoint modification sites, which are fundamental for understanding this modification and provide valuable information for future exploration of the functions of this modification. Here, we developed a method to systematically and site-specifically identify protein co-translational *O*-GlcNAcylation in human cells. Using the pulse-chase labeling using heavy amino acids, newly synthesized polypeptides were isotopically labeled in a short period of time, which were distinguished from the existing ones by MS. The Puro treatment generated premature polypeptide chains, ensuring that newly synthesized proteins were not folded, and post-translational modifications did not occur.

The abundance of the immediate translation products is normally very low in cells, and that of the co-translational *O*-GlcNAcylated form of nascent proteins is even much lower. This makes the detection of the co-translational modification extremely challenging. By integrating a boosting approach, it dramatically enhances the identification of co-translational *O*-GlcNAcylated peptides, enabling us to globally analyze protein co-translational *O*-GlcNAcylation. Previously, our laboratory used a boosting approach to study secreted glycoproteins in the serum-containing media. Some low-abundance secreted glycoproteins could be masked by highly abundant serum proteins even with the enrichment. If cells were cultured in the serum-free media, secreted proteins could be better detected, but cell starvation can dramatically alter protein secretion. To overcome these issues, our laboratory devised an approach to profile secreted *N*-glycoproteins in the serum-containing media using secreted glycoproteins from the serum-free medium as a boosting sample.^40^

In this work, we used cells labeled with Ac_4_GalNAz for 48 h to boost the detection of low-abundant co-translationally *O*-GlcNAcylated proteins that cannot be detected without the boosting, as demonstrated in the experiment without the boosting. The current method enables global and site-specific characterization of co-translational *O*-GlcNAcylation sites. In the boosting sample labeled with Ac_4_GalNAz for 48 h, it contains both mature glycoproteins and nascent polypeptides with co-translational *O*-GlcNAcylation. Therefore, any sites modified on nascent polypeptides are also in the boosting sample. Their signals are enhanced as well. However, the abundance of mature glycoproteins could be higher than co-translational glycopeptides. Therefore, it is possible that a small subset of nascent polypeptides with co-translational *O*-GlcNAcylation only occurring on them may not be detected. In total, 182 co-translational *O*-GlcNAcylated proteins were identified. In striking contrast, without the boosting approach, no co-translational glycopeptide was identified using the same workflow. In this work, HCD was used for *O*-GlcNAcylated peptide fragmentation. Despite that the glycan (*O*-GlcNAc) is small, the neutral loss could happen under HCD, affecting the site localization. To improve *O*-GlcNAcylation site localization, it would be better to use other fragmentation methods, such as electron-transfer dissociation (ETD) and electron-transfer/high-energy collision dissociation (EThcD).

Further bioinformatics analysis found that co-translational *O*-GlcNAcylated proteins are more enriched in the nucleus and associated with transcription compared with total *O*-GlcNAcylated proteins in the same cells. These results pose an intriguing question about a potential role of co-translational *O*-GlcNAcylation in gene transcription. Moreover, the adjacent residues and the secondary structures of co-translational *O*-GlcNAcylation sites are quite different from total *O*-GlcNAcylation sites, and it could be due to the preference of OGT for the neighboring residues of glycosylation sites and the accessibility of glycosylation sites when proteins are not well-folded.

In conclusion, we developed an effective method integrating selective enrichment, a boosting approach, and multiplexed proteomics to globally and site-specifically identify protein co-translational *O*-GlcNAcylation. The method differentiates co-translational glycopeptides from pre-existing ones through the pulse-chase labeling and puromycin treatment. SP3 sample preparation ensures the efficient capture of truncated glycopeptides. Co-translational *O*-GlcNAcylated peptides are of very low abundance in cells, making their detection extremely challenging. This is overcome by introducing a boosting approach using enriched *O*-GlcNAcylated peptides from the cells labeled with the sugar analogue for a much longer time, which contains both post-translational and co-translational *O*-GlcNAcylation. Over 180 co-translationally *O*-GlcNAcylated proteins were site-specifically identified. Compared with all *O*-GlcNAcylated proteins identified from the same cells, the co-translational ones have higher nuclear distribution and are more associated with transcription. The secondary structures and adjacent residues also have remarkable differences between the co-translational and total *O*-GlcNAcylation sites. The unprecedented information about protein co-translational *O*-GlcNAcylation can advance our understanding of this important modification.

## Data Availability

The raw files generated in this study were deposited to public accessible database MASSIVE (ftp://MSV000090569@massive.ucsd.edu).

## Supporting Information Available

The workflow to identify protein co-translational *O*-GlcNAcylation without the boosting sample (Figure S1); average TMT intensities of *O*-GlcNAcylated peptides quantified in this study and the extended view of the sum intensities of the TMT reporter ions for the Puro-treated and the control samples (Figure S2); comparison of the dynamics of proteins identified with co-translational glycosylation sites and others in the cytoplasm (Figure S3); comparison of the molecular weights of the total and co-translational *O*-GlcNAcylated proteins (Figure S4); comparison of the possibility of the total and co-translational *O*-GlcNAcylation sites next to phosphorylation or ubiquitination sites (Figure S5); and comparison of the possibility of the total and co-translational *O*-GlcNAcylation sites located in ordered and disordered regions (A) or in buried, medium, or exposed regions (Figure S6) (PDF)
Unique co-translational *O*-GlcNAcylated peptides identified in MCF7 cells (Table S1) (XLSX)
Results for the identification of total *O*-GlcNAcylated peptides from MCF7 cells (Table S2) (XLSX)
Unique co-translational *O*-GlcNAcylated peptides from transcription factors in MCF7 cells (Table S3) (XLSX)
Well-localized co-translational *O*-GlcNAcylation sites identified in MCF7 cells (Table S4) (XLSX)
Well-localized *O*-GlcNAcylation sites from MCF7 cells (Table S5) (XLSX)
