# Supplementary material for: Combining Selective Enrichment and a Boosting Approach to Globally and Site-Specifically Characterize Protein Co-translational O-GlcNAcylation
Source: Anal Chem. 2023 Feb 20;95(9):4371–80. doi: 10.1021/acs.analchem.2c04779 (PMC9996615; doi:10.1021/acs.analchem.2c04779)
Supplement: Supplementary file 1 — ac2c04779_si_001.pdf [file ac2c04779_si_001.pdf]

Supporting Information

Combining selective enrichment and a boosting approach to globally and site-specifically characterize protein co-translational O-GlcNAcylation

Senhan Xu, Kejun Yin, and Ronghu Wu*

School of Chemistry and Biochemistry and the Petit Institute for Bioengineering and Bioscience, Georgia Institute of Technology, Atlanta, Georgia 30332, USA

* Corresponding author: Phone: 404-385-1515; Fax: 404-894-7452,

Table of Contents

A list of Supporting Tables S2
Figure S1 .... S3
Figure S2 .... S4
Figure S3 .... S5
Figure S4 .... S6
Figure S5 .... S7
Figure S6 .... S8

A list of Supporting Tables in Excel format:

Table S1. Unique co-translational O-GlcNAcylated peptides identified in MCF7 cells (XLSX).

Table S2. Results for the identification of total O-GlcNAcylated peptides from MCF7 cells (XLSX).

Table S3. Unique co-translational O-GlcNAcylated peptides from transcription factors in MCF7 cells (XLSX).

Table S4. Well localized co-translational O-GlcNAcylation sites identified in MCF7 cells (XLSX)

Table S5. Well localized O-GlcNAcylation sites from MCF7 cells (XLSX)

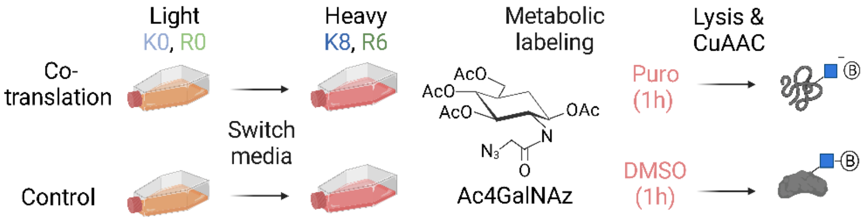

Figure S1. The workflow to identify protein co-translational O-GlcNAcylation without the boosting sample.

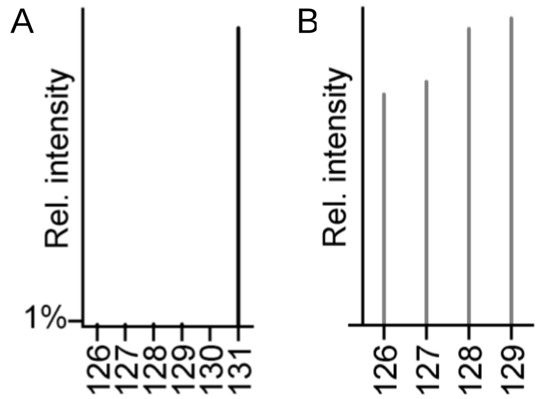

Figure S2. (A) Average TMT intensities of O-GlcNAcylated peptides quantified in this study. (B) The extended view of the sum intensities of the TMT reporter ions for the Puro treated and the control samples.

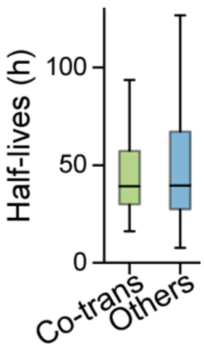

Figure S3. Comparison of the dynamics of proteins identified with co-translational glycosylation sites and others in the cytoplasm.

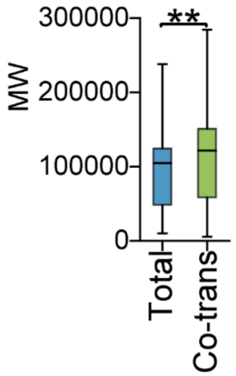

Figure S4. Comparison of the molecular weights of the total and co-translational O-GlcNAcylated proteins. In the boxplots: Center line, mean; Box limits, the first and third quartiles; Whiskers, 1.5 interquartile range. Statistical significance was determined by the Student's t-test, two tailed. The significance level is labeled as ** (P<0.01)

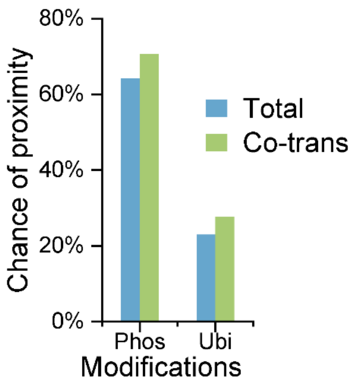

Figure S5. Comparison of the possibility of the total and co-translational O-GlcNAcylation sites next to phosphorylation or ubiquitination sites.

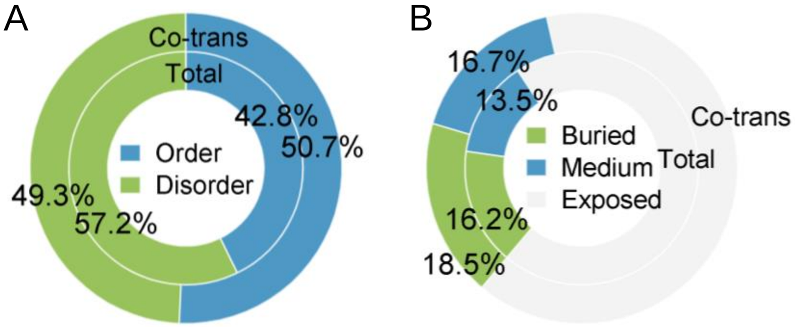

Figure S6. (A and B) Comparison of the possibility of the total and co-translational O-GlcNAcylation sites located in ordered and disordered regions (A), or in buried, medium or exposed regions (B).
